# Pyroligneous Acids of Differently Pretreated Hybrid Aspen Biomass: Herbicide and Fungicide Performance

**DOI:** 10.3389/fchem.2021.821806

**Published:** 2022-02-08

**Authors:** Pasi Korkalo, Marleena Hagner, Janne Jänis, Marko Mäkinen, Janne Kaseva, Ulla Lassi, Kimmo Rasa, Tuula Jyske

**Affiliations:** ^1^ Production Systems, Natural Resources Institute Finland (Luke), Rovaniemi, Finland; ^2^ Natural Resources, Natural Resources Institute Finland (Luke), Jokioinen, Finland; ^3^ Ecosystems and Environment Research Programme, University of Helsinki, Helsinki, Finland; ^4^ Department of Chemistry, University of Eastern Finland, Joensuu, Finland; ^5^ Research Unit of Sustainable Chemistry, University of Oulu, Oulu, Finland; ^6^ Production Systems, Natural Resources Institute Finland (Luke), Jokioinen, Finland; ^7^ Production Systems, Natural Resources Institute Finland (Luke), Espoo, Finland

**Keywords:** pyroligneous acid, hybrid aspen, biomass, torrefaction, biopesticide, herbicide, fungicide, cascade utilization

## Abstract

The pyroligneous acids (PAs) of woody biomass produced by torrefaction have pesticidal properties. Thus, PAs are potential alternatives to synthetic plant protection chemicals. Although woody biomass is a renewable feedstock, its use must be efficient. The efficiency of biomass utilization can be improved by applying a cascading use principle. This study is novel because we evaluate for the first time the pesticidal potential of PAs derived from the bark of hybrid aspen (*Populus tremula* L. × *Populus tremuloides* Michx.) and examine simultaneously how the production of the PAs can be interlinked with the cascade processing of hybrid aspen biomass. Hybrid aspen bark contains valuable extractives that can be separated before the hemicellulose is thermochemically converted into plant protection chemicals. We developed a cascade processing scheme, where these extractives were first extracted from the bark with hot water (HWE) or with hot water and alkaline alcohol (HWE+AAE) prior to their conversion into PAs by torrefaction. The herbicidal performance of PAs was tested using *Brassica rapa* as the test species, and the fungicidal performance was proven using *Fusarium culmorum*. The pesticidal activities were compared to those of the PAs of debarked wood and of commercial pesticides. According to the results, extractives can be separated from the bark without overtly diminishing the weed and fungal growth inhibitor performance of the produced PAs. The HWE of the bark before its conversion into PAs appeared to have an enhancing effect on the herbicidal activity. In contrast, HWE+AAE lowered the growth inhibition performance of PAs against both the weeds and fungi. This study shows that hybrid aspen is a viable feedstock for the production of herbicidal and fungicidal active chemicals, and it is possible to utilize biomass according to the cascading use principle.

## Introduction

One possible way to produce acidic chemical products from biomass is thermochemical conversion, e.g., torrefaction (Fagernäs et al., 2015; Grewal et al., 2018). Typically, the technique is used as a biomass pretreatment process, such as for gasification or combustion (Van der Stelt et al., 2011; Cahyanti et al., 2020). Torrefaction is a technique similar to slow pyrolysis, where conversion is performed at a slow heating rate under anaerobic conditions, but is conducted at lower temperatures. When using lower process temperatures, the hemicelluloses of lignocellulosic biomass are the most essential precursors to produce pyroligneous acids (PAs) (Collard and Blin, 2014; Chen et al., 2019). PAs are known to have pesticidal activity (Oramahi and Yoshimura, 2013; Theapparat et al., 2015; Oramahi et al., 2018; Hagner et al., 2020); therefore, the method appears to be a viable way to produce bio-based pesticides as a substitute for synthetic chemicals (Tiilikkala et al., 2010). Wood material is a potential and renewable natural resource for the production of pesticide-active chemicals, but the sustainable use of forest resources must be taken into account (Cambero and Sowlati, 2014). This can be promoted by studying the use of raw materials according to the cascade principle and by investigating the effects of the processes on the chemical products.

**FIGURE 1:**
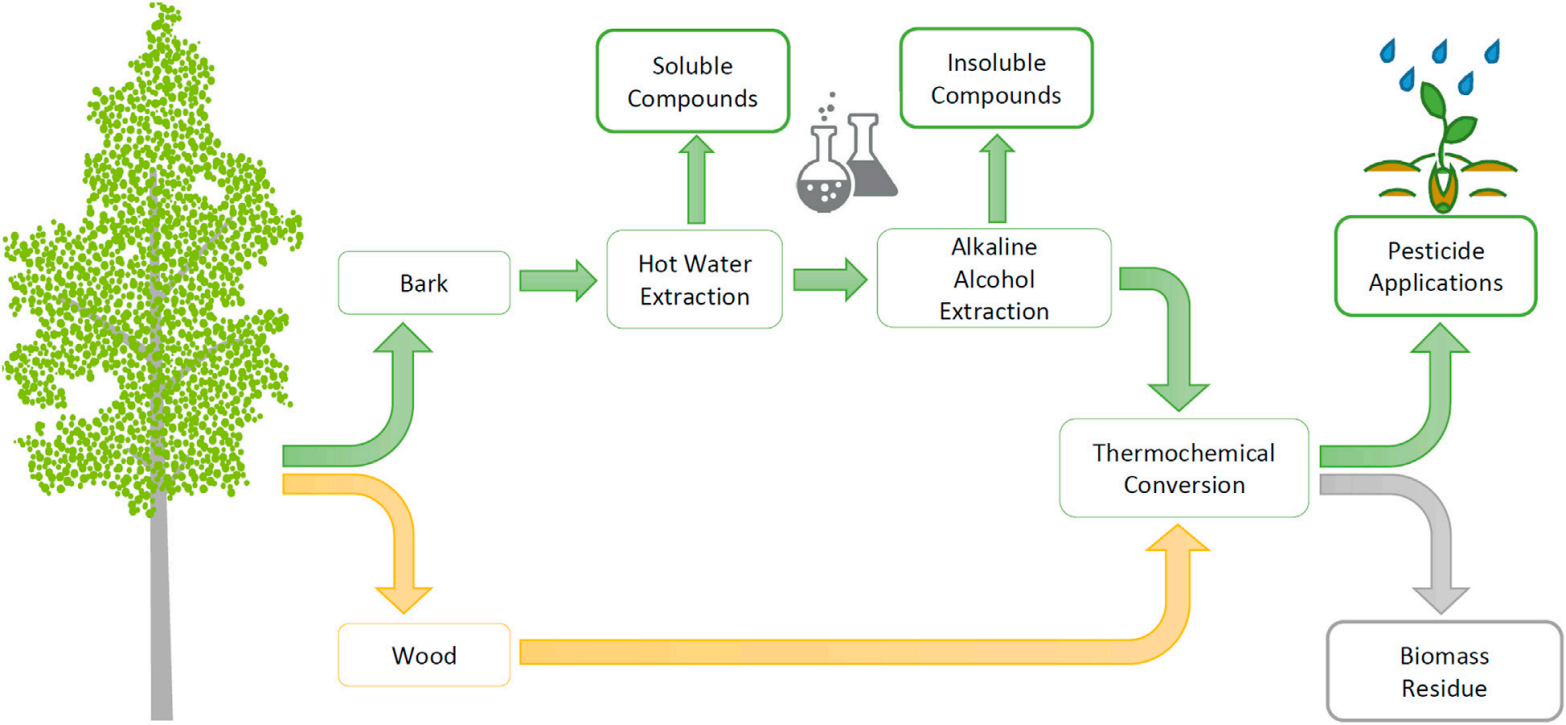
Utilization of hybrid aspen biomasses in the production of biopesticides according to the cascade principle.

In recent years, the utilization of biomass in chemical production has increased. Recovery techniques are mainly conversion processes, such as catalytic, biological, and thermochemical processes. So far, the sources of bio-based raw materials for biochemical production are largely those that compete with other sectors, such as the food industry. In the cultivation of forest as a feedstock source, such a competitive situation is more easily evaded (Cherubini, 2010). Currently, the high market potential for bio-based chemicals includes biofuels, biomaterials such as degradable plastics, and a variety of biochemical applications such as adhesives and packaging coatings (Cherubini, 2010; Hassan et al., 2019). The number of bio-based products on the market is clearly growing, but the utilization of by-products is still low (Cherubini, 2010). As the chemical industry aims to electrify processes with renewable energy, produce biochemicals by converting from forest biomass, and reduce the need for fossil-derived reagents in biochemical production (Toivanen et al., 2021), pyrolysis techniques such as torrefaction are essential methods to be explored. The method also has the advantage of being able to generate side streams for recovery (Figure 1).

There are still numerous encounters with the enhanced use of forest biomass, such as availability, price, and sustainability (Toivanen et al., 2021). One possible solution could be to cultivate fast-growing tree species as a source of raw materials for chemical production. By cultivating clonal trees, the raw material can be tailored (Korkalo et al., 2020) to serve better the needs of the chemical industry, locate the biomass source closer to chemical production, and accelerate biomass production (Beuker et al., 2016).

At the same time, as the goal is to increase the utilization of forest biomass in the chemical industry, the adequacy and sustainable consumption of forest resources must be taken into account (Azapagic, 2014). This means more efficient and versatile use of raw materials, where cascade utilization plays a key role. The term “cascade utilization” describes an enhanced use of biomass that produces multiple products before the biomass ends up as a waste residue, energy production, or other end-products. There is no consistent description of the concept of the cascade principle, but the definition of the term is met if the utilization of biomass involves linear utilization, product side streams, recycling, and other activities that increase the life cycle of biomass or maximizes its value in use (Keegan et al., 2013; Sokka et al., 2015).

Cultivated hybrid aspen is an interesting raw material alternative for various bio-based conversion products due to its rapid biomass yield potential. In Finland, the tree species reaches its felling age in the first growth cycle in 20–25 years and in the second growth cycle in 15 years. In northern growth areas, the growth rate compared to other commercially important tree species can be 40% faster than birch and 50% faster than spruce. Thanks to its rapid growth, the biomass yield of cultured hybrid aspen can reach up to 20 m^3^ ha^−1^ year^−1^ in 25-year cycles (Beuker et al., 2016).

This study investigated the use of hybrid aspen (*Populus tremula* L. × *Populus tremuloides* Michx) as a feedstock and the cascade utilization of the biomass in the production of herbicidal and fungicidal active chemicals (Figure 1). Due to the low extractive content of the debarked wood, the wood biomass is led directly to thermochemical conversion. However, the bark is known to be rich in extractives (Korkalo et al., 2020), so the effects of bark raw material pre-extraction on the pesticidal activity of PAs were studied.

Hot water extraction (HWE) was chosen as the first pre-extraction step for bark cascade processing (Figure 1) since the method can be used to separate soluble hydrophilic extractives into value-added products (Rasi et al., 2019). Moreover, HWE at moderate temperature and normal atmospheric pressure is not expected to significantly affect bark hemicellulose contents. The soluble hydrophilic compounds consist of tannins and strongly antioxidant phenolic substances (Korkalo et al., 2020). These groups of compounds are of interest for several practical applications, e.g., tannins in rigid foam forming (Tondi and Pizzi, 2009; Varila et al., 2020) and phenolic compounds as antioxidants in various end uses (Reuter et al., 2010).

Alkaline alcohol extraction (AAE), which can be used to separate insoluble bark compounds such as suberinic acids, was chosen as the second pre-extraction step to be tested (Figure 1). Suberin-derived fatty acids have been found to have promising properties to act, e.g., as a coating material for paper, fabrics, and packaging (Korpinen et al., 2019). The pretreatment of the bark with AAE prior to conversion to the biopesticide has been considered, even though hemicellulose structures are known to be soluble under alkaline conditions (Stoklosa and Hodge, 2012). Since hemicelluloses are the essential precursors for PA formation, the effect of bark AAE pretreatment on the pesticidal activity of PAs was analyzed in this study.

The hypothesis of this study is that several valuable products can be separated from hybrid aspen biomass without losing all the potential of PAs to be suitable for herbicide and fungicide applications. The herbicidal activity of PAs produced from wood, bark, and pre-extracted bark was tested using *Brassica rapa* as the model plant and the fungicidal activity tested using *F* *usarium culmorum*. The activity tests examined how well the bio-based pesticides compete with the efficacy of commercial products and whether the biomass can be utilized in the production of biopesticides according to the cascade principle.

## Materials and Methods

### Chemical Analysis of Wood and Bark Biomass

#### Samples

Six sample trees of hybrid aspen clones (*P. tremula* L. × *P. tremuloides* Michx.) were randomly selected from a designed field trial of the Natural Resources Institute Finland (Luke) near Lohja, Southern Finland (60°12′ N, 23°55′ E). For chemical analyses of hybrid aspen biomass, a sample disc was cut from each tree at breast height (1.3 m). Bark mass, including both inner and outer layers, was peeled from the wood, after which both separated sample fractions were freeze dried. Dried bark and wood samples were ground with a 0.75 mm milling blade (rotor mill PULVERISETTE; FRITSCH, Idar-Oberstein, Germany) and stored at −20°C until analysis. Chemical assays made from wood and bark were used to characterize the wood chemical composition of hybrid aspen biomass and to examine the differences between the separated biomass fractions. Chemical characterizations were determined by performing similar chemical analyses for both sample fractions, except for suberinic acids which were determined only from bark samples (Table 1).

**TABLE 1:** Basic information on hybrid aspen tree samples, chemical composition analysis for the wood and bark biomass fractions, number of biomass samples prepared for torrefaction, and chemical characterization of pyroligneous acids

Hybrid aspen clone sample tree information	
*Populus tremula* L. × *Populus tremuloides* Michx.	*n* = 6
Planting year	1998
National Register ID^a^	C05-99-14
Log volume (m^3^)	0.571 ± 0.068
Height (dm)	278 ± 10
Diameter 1.3 (mm)	236 ± 22
Diameter 5.0 (mm)	216 ± 19
Bark yield, fresh weight (wt%)	6.5 ± 0.4
Chemical analyses of the hybrid aspen wood and bark samples	
Tree trunk sampling height	1.3 m
No. of bark and wood samples	*n* = 6
Hydrophilic extractives: *wood and bark*	Gravimetric analysis
Lipophilic extractives: *wood and bark*	Gravimetric analysis
Suberin-derived fatty acid: *bark*	GC-MS
Lignin: *wood and bark*	Soluble and insoluble lignin
Cellulose: *wood and bark*	Acid hydrolysis, GC-FID
Hemicellulose: *wood and bark*	Acid methanolysis, GC-FID
Hybrid aspen feedstock samples for torrefaction	
Wood	*n* = 1
Bark	*n* = 1
HWE bark	Hot water extracted, *n* = 1
HWE+AAE bark	Hot water and alkaline alcohol extracted, *n* = 1
Chemical analyses of pyroligneous acids	
Acid content	Titrimetric determination
Water content	Karl Fischer titration
Organic matter	Calculated based on acid and water contents
Compositional analysis of organic matter	FT-ICR characterization

*HWE*, hot water extraction; *AAE*, alkaline alcohol extraction; *GC-FID*, gas chromatography with a flame ionization detector; *FT-ICR*, Fourier transform ion cyclotron resonance

a The national list of approved basic forest reproductive material, kept by the Finnish Food Authority (Finnish Food Authority, 2021).

For the torrefaction and the bark cascade processing experiments, wood, bark, hot water-extracted bark (HWE bark), and hot water and alkaline alcohol-extracted bark (HWE+AAE bark) samples were prepared by pooling the biomass of six sample trees into representative bulk samples of each feedstock. Feedstock samples were prepared for the torrefaction experiments freshly after collection. For the pesticide (i.e., herbicide and fungicide) experiments, one PA sample was produced from each of the feedstocks. The collected PA samples were used for pesticide experiments and for acid strength analysis shortly after the conversion. Information on the hybrid aspen tree, sampling data, and the list of chemical analyses are shown in Table 1.

#### Lipophilic and Hydrophilic Extractive Contents

Lipophilic and hydrophilic extractives of wood (*n* = 6) and bark (*n* = 6) were determined from freeze-dried and finely ground samples using the accelerated solvent extraction (ASE-350, Dionex, Sunnyvale, CA, USA) method. Lipophilic compounds were extracted with hexane at 90°C three times for 15 min, followed by extraction of the hydrophilic compounds with 95% acetone (aq) at 100°C three times for 15 min. Each separated lipophilic and hydrophilic extractive solution was adjusted to a final volume of 50 ml with used extractants, after which samples of each solution were dried in a nitrogen evaporator at 40°C to a dry matter residue to determine the extractive yields. Extract-free wood and bark samples were used for the determination of hemicellulose, cellulose, lignin, and suberinic acid contents of the bark. The method was modified from Willför et al. (2003).

#### Determination of Bark Suberinic Acid

The suberinic acid content of the bark was determined from extractive-free samples (*n* = 6) with a modified method adapted from Krogell et al. (2012). Bark samples were weighed into seal tight test tubes, followed by the addition of 3% potassium hydroxide–ethanol solution (KOH, *w*/*v*). The bark samples were extracted for 2 h at 70°C. The solution containing suberinic acids was separated and collected by vacuum filtration. The bark residue was dried overnight at 105°C and saved for lignin assays. Suberinic acid solution samples were measured into sample tubes and diluted with water. Bromocresol green was added to the samples as a pH indicator, after which the sample solutions were acidified with 0.25 M aqueous sulfuric acid. After pH adjustment, an internal standard mix of C21:0 and betulinol in *tert*-butyl methyl ether (MTBE) was added to the samples. Suberinic acids, including the internal standard, were separated from aqueous solution using liquid–liquid extraction with the MTBE solvent. The liquid–liquid extraction was repeated a total of three times. Finally, the collected suberinic acid and the internal standard containing the MTBE solution was washed with water before drying in a nitrogen evaporator at 40°C. The dry residues of the samples were silylated by adding a reagent mix of pyridine–BSTFA–TMCS in a 1:4:1 ratio and allowed to react at 70°C for 45 min. The clear phase of the silylated samples was collected and the content of suberinic acids was quantitated against the internal standard of C21:0 using gas chromatography–mass spectrometry (GC-MS).

#### Determination of Lignin Content

The lignin content of the hybrid aspen samples was determined for wood from the extractive-free samples (*n* = 6) and for the bark from samples previously prepared for the determination of suberinic acids (*n* = 6). The total lignin content of the samples was determined as the sum of acid-soluble and insoluble (Klason) lignin. The prepared bark and wood samples were dried at 105°C overnight and weighed in duplicate for analysis. Of sulfuric acid, 72% was added to the sample vessels and mixed thoroughly, after which the samples were incubated for 1 h at 30°C. After incubation, the acid content of the samples was diluted to 4% with water, and the solution was transferred into a glass vessel with a screw cap. The samples were then placed in an autoclave at 120–125°C for 1 h, after which they were removed from the autoclave and allowed to cool to room temperature. Acid-insoluble lignin was separated from the samples using a vacuum filter and finally dried overnight at 105°C for weighing. The acid-soluble lignin separated by vacuum filtration was analyzed spectrophotometrically at 240 nm. The lignin content was analyzed and calculated according to the laboratory analytical procedure (LAP) (Sluiter et al., 2012).

#### Determination of Cellulose Content

The cellulose content of bark (*n* = 6) and wood (*n* = 6) was determined from the extract-free samples with the acid hydrolysis method adapted from Krogell et al. (2012). Samples and cellulose standards were weighed into sealable glass tubes, followed by the addition of 72% aqueous sulfuric acid. The strong acid solution was allowed to take effect for 2 h, after which it was diluted with water and allowed to react for the next 4 h. The acid content of the samples was further reduced by the addition of water and the samples left to stand overnight at room temperature. The following day, the samples were placed in an autoclave for 1 h at 120–125°C. Bromocresol green was added as a pH indicator, and the acidity of the solutions was neutralized with barium carbonate. d-Sorbitol was added into the wood, bark, and standard samples as an internal standard, after which the samples were mixed thoroughly and centrifuged. The separated clear phase was collected and evaporated to dryness in a nitrogen evaporator at 40°C, and finally in a vacuum oven at 40°C. Samples were silylated by the addition of pyridine, hexamethyldisilazane (HMDS), and trimethylchlorosilane (TMCS) and left to react overnight. The next day, the samples were analyzed by gas chromatography with a flame ionization detector (GC-FID). The cellulose contents of the samples were calculated by means of an inner standard.

#### Determination of Hemicelluloses Content

The acid methanolysis method for the determination of bark (*n* = 6) and wood (*n* = 6) hemicelluloses content was adapted from Sundberg et al. (1996). Extract free bark and wood samples, as well as standard samples (monosaccharide mix of arabinose (Ara), glucose (Glc), glucuronic acid (GlcA), galactose (Gal), galacturonic acid (GalA), mannose (Man), rhamnose (Rha), 4-*O*-methylglucuronic acid (4-*O*-Me-GlcA), and xylose (Xyl); 1.0 mg/ml of each) were measured in sealable pressure-resistant glass containers. Methanolysis reagent (2 M HCl in anhydrous methanol) was added to the samples and placed in a 100°C oven for 5 h. After cooling the samples, the acid content of the samples was neutralized with pyridine, and a mixture of sorbitol and resorcinol in methanol was added to the samples as an internal standard. The separated clear phase was collected from the samples and dried in a nitrogen evaporator at 40°C to dryness. Samples were silylated by the addition of pyridine, HMDS, and TMCS and left to react overnight at room temperature. The samples were then analyzed with GC-FID to identify hemicellulose sugar units and to calculate the concentrations by using internal standards to determine the hemicellulose content.

### Biomass Pre-Extraction and Torrefaction Process to Produce Plant Protection Chemicals

#### Preparation of a Hot Water-Extracted Bark Sample for Torrefaction

To prepare the HWE bark feedstock sample (*n* = 1), 2 kg of bark mass was pooled from six sample trees. For the extraction, the water-to-dry matter ratio was adjusted to 6.1:1 (*v*/*m*) by checking the dry matter (d.m.) content of the bark sample with a moisture analyzer (MLB 50-3N, KERN & Sohn GmbH, Balingen, Germany) before adding the required amount of water. Bark mass was extracted in a steel boiler at normal atmospheric pressure at 90°C for 2 h. After extraction, the HWE bark sample was divided into two: one half was saved for torrefaction and the other half saved for the following alkaline alcohol extraction (Figure 1).

#### Preparation of the Alkaline Alcohol-Extracted Bark Sample for Torrefaction

The hot water and alkaline alcohol-extracted bark (HWE+AAE bark, *n* = 1) was prepared from the previously prepared HWE bark mass. The AAE method was adapted from Rižikovs et al. (2014) and Korpinen et al. (2019). The AAE extractant mixture was prepared from crystalline sodium hydroxide (NaOH), water, and IPA (2-propanol) (Merck KGaA, Darmstadt, Germany) in a ratio of 1,200 ml IPA:300 ml H_2_O:20 g NaOH:100 g HWE bark d.m. The amounts of NaOH, water, and IPA required for the extractant mixture were calculated based on the d.m. content of the HWE bark sample, which was checked with a moisture analyzer (MLB 50-3N, KERN & Sohn GmbH, Balingen, Germany). The extraction solvent was prepared by dissolving NaOH in water before mixing with IPA and finally adding with HWE bark into a glass laboratory flask. The sample was extracted at normal atmospheric pressure by heating the extraction vessel in a hot water bath at 85°C for 3 h. After extraction, the HWE+AAE bark sample was rinsed with water until the pH of the rinsing water decreased.

#### Torrefaction of Wood, Bark, and Pre-Extracted Bark Biomass

The biomass conversion process was carried out in a bench-scale slow pyrolysis batch reactor under anaerobic conditions. The oxygen in the system was removed by purging the pyrolysis vessel with nitrogen gas before heating. The pyrolysis equipment consisted of a temperature control unit, an indirectly heating furnace, a gas-tight pyrolysis vessel with an internal thermometer (TCC-K250-6.0-KY), a water-cooled condenser with a condensate collection vessel, and a gas flow meter (drum-type gas meter, TG1/5, RITTER, Bochum, Germany). Hybrid aspen wood, bark, HWE bark, and HWE+AAE bark feedstocks were dried at 37°C before being placed into the pyrolysis apparatus. The sample sizes and d.m. contents (in weight percent), respectively, of dried biomass were 440.1 g, 97.7 wt.% for wood; 440.0 g, 96.2 wt.% for bark; 440.0 g, 96.0 wt.% for HWE bark; and 231.0 g, 96.2 wt.% for HWE+AAE bark. The torrefaction heating steps were programmed to ramp up from the pre-drying phase to the target temperature of 280°C. The actual measured heating rate of the pyrolysis vessel was 1.7°C/min from an initial temperature range of 30–250°C, after which the heating rate was decreased before reaching the temperature of 279 ± 2°C. The level of the target temperature was maintained for 40–50 min. The yields of solids and PAs formed during torrefaction were weighed and the total mass of gas formed calculated by subtracting the total mass of the solid and PA products from the weights of the feedstock samples loaded. All product yields were determined as mass percentages (weight percent) of the amount of samples loaded into the pyrolysis vessel.

### Chemical Analysis of Conversion Distillates

#### Pyroligneous Acid Titratable Acidity

The titratable acid strength of the PAs was determined by titrating the solutions with 1 M aqueous NaOH solution and calculating the result as acetic acid equivalent. Titration pH change was measured with a pH meter (SevenExcellence, Mettler-Toledo, Columbus, OH, USA) and electrode (Mettler-Toledo InLab Expert Pro-2m-ISM). The acetic acid equivalence of the condensates was determined by plotting the change in pH with the consumption of NaOH, verifying the titration equivalence point with the first (ΔpH/Δ*V*) and second (ΔpH^2^/Δ^2^ *V*) derivatives, and finally calculating the titratable acid content of the solutions as acetic acid CH_3_COOH (% *m*/*v*) equivalence.

#### Water and Organic Matter Contents of Condensates

The water contents of the condensed liquids were determined with Karl Fisher titration using the volumetric ASTM E203-08 method. The measurements were made with the Metrohm 870KF Titrino Plus titrator equipped with double Pt-wire electrode (Metrohm AG, Herisau, Switzerland). The titrant was the commercial Hydranal Composite 5K reagent (Sigma-Aldrich, St. Louis, MO, USA). A mixture of chloroform and methanol (3:1, *v*/*v*) was used as the solvent. The result of each sample was reported as the average of three parallel measurements. The proportion of organic matter in pyroligneous acids was determined by subtracting the water and the titratable acid contents from the total mass of the liquid.

#### Compositional Analysis of Organic Matter

A further compositional analysis of the organic matter in the conversion distillates was performed using ultrahigh-resolution direct-infusion mass spectrometry, which allows a non-targeted complex mixture characterization without chromatographic separation. All the measurements were performed on a 12-T solariX XR Fourier transform ion cyclotron resonance (FT-ICR) mass spectrometer (Bruker Daltonics GmbH, Bremen, Germany) using negative-ion electrospray ionization (ESI). This instrument has been described in detail elsewhere (Zhao et al., 2020). The distillates were diluted with methanol at a ratio of 1:1,000 (*v*/*v*) and directly infused into the ion source with a syringe pump, operating at a flow rate of 2 μl/min. Dry nitrogen was used as the drying (200°C, flow rate of 4 L/min) and nebulizing gas (1 bar). The calibration of the mass spectra was done externally using sodium trifluoroacetate clusters. The ions were detected within a mass range of *m*/*z* 98–1,000. Three hundred time–domain transients were co-added for each spectrum with a data size of 4 MWord. Bruker Compass ftmsControl 2.1 software was used for the instrument control and data acquisition, and Bruker DataAnalysis 5.1 software was used for data post-processing. To improve mass accuracy, the mass spectra were further internally recalibrated with selected analyte ions. The signal-to-noise ratio was set at 5.0 for the peak picking, and the relative intensity threshold was 0.01%. The following parameters were used for the molecular formula assignment: elemental formula, ^12^C_1–100_^1^H_1–2000_^14^N_0–2_^6^O_0–25_^32^S_0–1_; double bond equivalent (DBE), 0–80; and mass error, ≤0.8 ppm. Microsoft Excel (Microsoft Corporation, Redmond, WA, USA) and CERES viewer 1.82 were used for the data sorting and visualization.

### Pesticidal Tests

#### Herbicidal Activity Test

In the herbicide tests, turnip rape (*B. rapa*; Apollo, Boreal Plant Breeding Ltd.) was used as the test species due to its recommended use in phytotoxicity assessments (OECD, 2006). The herbicidal effect of the PAs was assessed according to Hagner et al. (2020) using a Jacobsen germinator (Rubart Apparate GmbH, Laatzen, Germany) that consists of a germination plate being temperature-conditioned by means of a water basin below. Germination spirals (filter papers, *Ø* = 6 cm) equipped with a paper wick were placed on the germination plate. The wick was led through slots in the germination plate into the water basin below, thus supplying the required humidity and temperature (20°C) to the germination spiral. Turnip rape seeds (20 pieces) were placed on the germination spirals, which were covered with a transparent glass dome to provide the air humidity required for germination. A little hole in the dome ensured minimum evaporation. Seeds achieved 16-h light a day. After 7 days, the covers were removed, the germinated seedlings were counted, and the germination spirals with seedlings were gently transferred into Petri dishes for herbicide treatments. An even deposit of the test liquid over the seedlings was achieved using a Potter precision laboratory spray tower spraying 1.0 ml of the selected liquid per filter paper. Tap water was used as an inert control and BioNeko (120 g/L acetic acid) as the active control. The concentrations of the PAs tested were 50%, 25%, 12.5%, and 5% (*v*/*v*), each with four replicates. The treated germination spirals were returned into the Jacobsen germinator and the cover domes were set on their places. The number of living seedlings was counted after 7 days.

#### Fungicidal Activity Test

The fungicidal properties of the tested PAs were estimated using *F. culmorum* as the test species. The utilized *F. culmorum* was separated in 2005 from barley (*Hordeum vulgare* strain: *Presitge*) grown in Sotkamo, Finland, and stored in liquid nitrogen (−190°C) since then. Each test liquid was examined in the concentrations of 100%, 50%, 25%, 10%, and 5% (*v*/*v*). Tap water was used as an inert control and 1.5 g/L Switch (active substances: 375 g/kg cyprodinil and 250 g/kg fludioxonil) as an active control. Each treatment had three replicates. The test was conducted in Petri dishes containing 25 ml of potato dextrose agar (PDA) as growth media for fungus. Four filter papers (6 mm diameter) treated with 100 µm of the test liquid were placed on the growth medium of each Petri dish. The fungus was inoculated in the middle of the Petri dish and the lids closed. The dishes were incubated at 20°C. After 2, 4, and 7 days of incubation, the fungal-covered area was measured.

#### Statistical Analysis

All dependent variables (percentage of dead *B. rapa*, the *F. culmorum*-covered area, and the development of fungal coverage) were analyzed using generalized linear models (GLMs) with treatment (BioNeko/Switch, wood, bark, HWE bark, HWE+AAE bark, and water control), concentration (from 5% to 50%), and their interaction as fixed effects. The concentrations of the first model were 5%, 12.5%, and 20%, and those of the second were 5%, 10%, 25%, and 50%. In the latter model, a concentration of 25% was chosen and the measurement day (2, 4, and 7 days) was used as a fixed effect instead of concentration. Four replicates were used without randomization.

The assumption of beta distribution with a logit link was used for percentages (model 1), and for coverage areas, the assumptions of log-normal distribution with an identity link (model 2) and gamma distribution with a log link (model 3) were used because the distributions of areas were highly skewed. The restricted maximum likelihood (REML) estimation method was used for the second model, maximum likelihood (ML) for the first model, and residual pseudo-likelihood (REPL) for the third model.

Heterogeneous variances for treatments were allowed in the second model. In the third model, correlations between measurement days within the same experimental unit were taken into account using the unstructured covariance structure, which estimates unique correlations for each pair of time points. Both solutions were based on lower values of the information criterion (corrected Akaike information criterion, AICc), although a likelihood test was also used for the decision of heterogeneous variances for treatments.

All treatments were compared within each concentration (models 1 and 2) or measurement days (model 3). The step-down method of Westfall, which is known to be one of the most effective when the design is balanced, was used for the pairwise comparisons of means with a significance level of 0.05 (Westfall, 1997). The method of Kenward and Roger was used for calculating the degrees of freedom for models 2 and 3 and the residual method used for the first model (Kenward and Roger, 2009).

The analyses were performed using the GLIMMIX procedure of the SAS Enterprise Guide 7.15 (SAS Institute Inc., Cary, NC, USA).

## Results and Discussion

We hypothesized that several valuable products can be separated from hybrid aspen biomass without losing all the potential of PAs suitable for herbicide and fungicide applications. To prove this, the herbicidal and fungicidal activities of PAs produced from 1) wood, 2) untreated bark, 3) HWE-treated bark, and 4) HWE+AAE-treated bark were examined. The activity tests showed that aspen wood and bark can be used as raw materials for bio-based pesticides as the herbicidal and fungicidal activities of undiluted PA were comparable to those of commercial products. In addition, the bark biomass can be utilized in the production of biopesticides according to the cascade principle as HWE pretreatment even improved the herbicidal activity of bark PA.

### Basic Composition of Hybrid Aspen Biomass Fractions

The chemical characterization of the bark and wood of hybrid aspen trees was used to evaluate the potential of the raw material in terms of cascade processing and to consider the composition of the biomass in relation to the yield of PAs. In the chemical compositions of wood and bark (Table 2), the most significant difference in terms of cascade use can be found in the extractive contents. The bark was found to have a significantly higher content of hydrophilic extractives than wood, bark biomass thus having more potential as feedstock for cascade utilization. Due to its low hydrophilic extractive content, wood is best suited for direct conversion to PAs without pre-extractions. The content of lipophilic extractives in both biomass fractions was low. Thus, the separation of lipophilic compounds from wood or bark before conversion to PAs does not seem a promising way to increase the value of biomass. Variations in the cellulose and lignin contents were also found between wood and bark, but the differences were not considered essential as pesticidal active PAs are converted at ≤280°C, i.e., below the temperature where cellulose and lignin are thermally degraded (Collard and Blin, 2014).

**TABLE 2:** Basic wood chemical composition of the wood and bark biomass fractions (mean ± SD, *n* = 6) of *Populus tremula* L. × *Populus tremuloides* Michx.

Basic composition	Wood	Bark
Lipophilic extractives (mg/g)	7.7 ± 2.6	27.3 ± 7.1
Hydrophilic extractives (mg/g)	20.9 ± 3.3	168.0 ± 66.5
Suberin-derived fatty acids (mg/g)	NA	23.7 ± 1.6
Cellulose (mg/g)	501.1 ± 19.9	200.2 ± 5.8
Hemicellulose (mg/g)	223.3 ± 8.2	300.4 ± 18.9
Lignin total^a^(mg/g)	229.9 ± 5.4	266.4 ± 15.5
Acid-insoluble lignin (mg/g)	166.6 ± 3.8	223.6 ± 14.3
Acid-soluble lignin (mg/g)	63.6 ± 4.5	42.8 ± 2.8

^a^Total lignin content is the sum of acid-insoluble and acid-soluble lignin.

**FIGURE 2:**
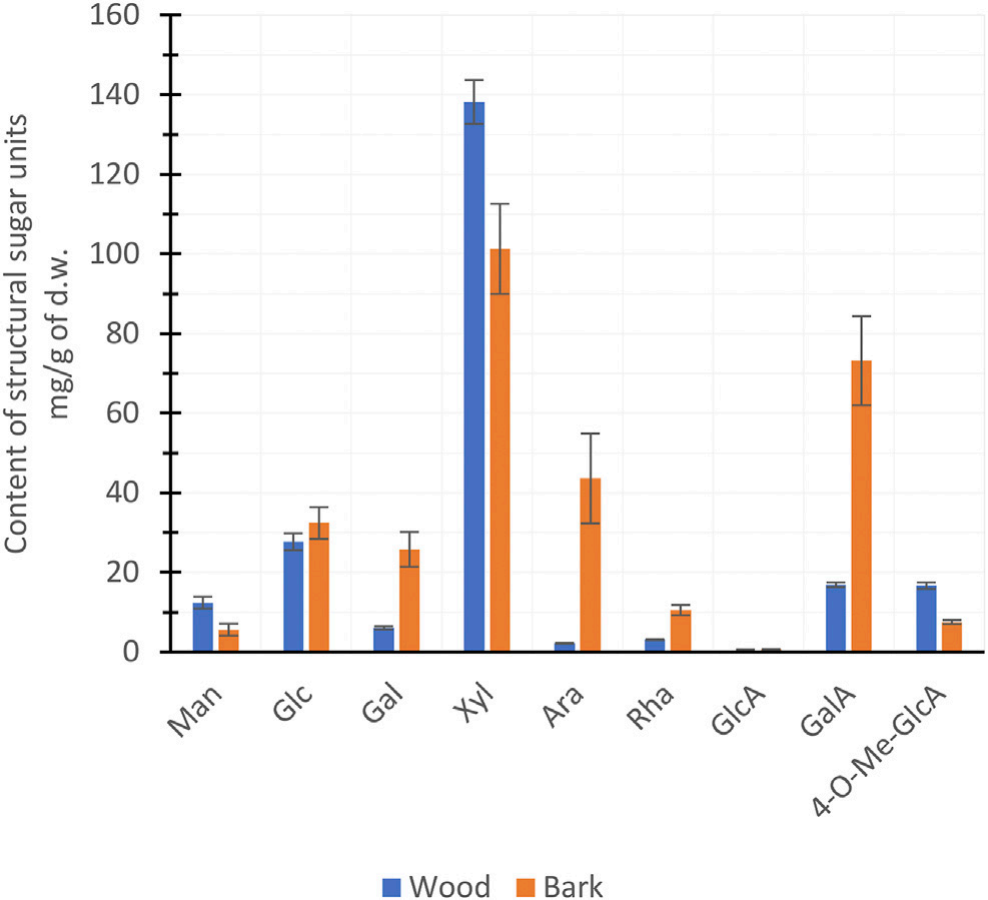
Mean (±SD) hemicellulose sugar units of hybrid aspen wood and bark per biomass dry weight (d.w.).

In the torrefaction of lignocellulosic biomass at ≤280°C, hemicelluloses are the most important precursors in PA production (Collard and Blin, 2014). The hemicelluloses of hardwood species consist mainly of xylan and, to a lesser extent, glucomannan and pectin (Sjöström, 1993; Willför et al., 2005). The chain structure of xylan consists of xylose (Xyl) units with an *O*-acetyl group attached to 7 units out of 10 and 4-*O*-Me-GlcA to 1 unit out of 10 in ratios (Sjöström, 1993). The *O*-acetyl groups of xylans are precursors of the acetic acid formed in thermochemical conversion; therefore, a proportion of xylan is the most decisive hemicellulose characteristic of biomass (Chen et al., 2019). The xylan content of the wood and bark of hybrid aspen was estimated from the concentrations of the Xyl and 4-*O*-Me-GlcA sugar units (Figure 2). The bark was found to have a higher total amount of hemicellulose (Table 2), but a lower xylan content compared to the wood fraction (Figure 2). Due to its higher xylan content, wood can be considered as a more promising feedstock for the production of PAs with a stronger acid concentration.

### Torrefaction Process and Pyroligneous Acid Formation

#### Torrefaction Product Yields

**FIGURE 3:**
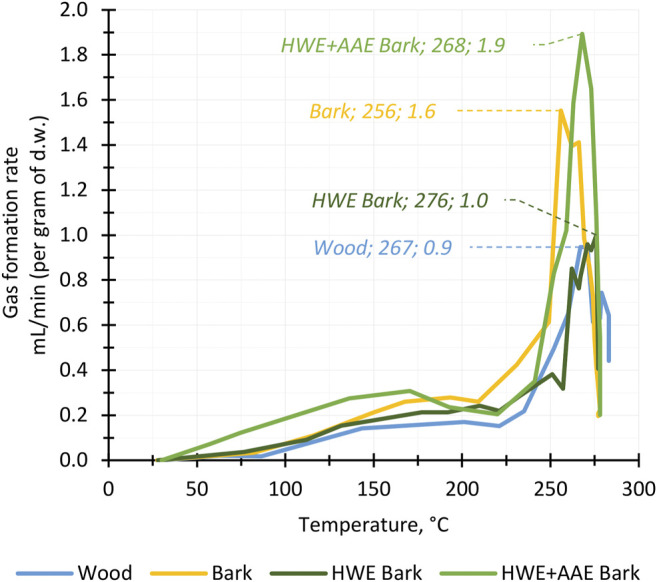
Gas production of hybrid aspen biomass fractions and pre-extracted bark masses (*HWE*, hot water extraction; *AAE*, alkaline alcohol extraction) during the torrefaction process. The maximum gas formation rate for each feedstock at different temperatures is highlighted in the diagram.

The formation of torrefaction products was used to examine the behavior of wood, bark, and pre-extracted bark during the conversion process. In torrefaction, the reactive temperature range for lignocellulosic biomass conversion begins at 150–175°C, and the actual conversion of hemicellulose occurs between 200°C and 300°C (Basu, 2013; Collard and Blin, 2014). The graph of the biomass gas formation rate during conversion showed that the reactive phase of hybrid aspen bark, wood, and pre-extracted bark biomasses began above 220°C and was at its most active phase above 250°C (Figure 3). Bark pre-extractions did not appear to have a significant effect on the reactive temperature range as all biomasses produced gas over the same temperature range. Although there were differences in the gas formation rates between the different biomasses, the total amounts of gases produced were only a few percentage points apart and did not appear to follow a clear trend relative to bark cascading pretreatments. For all feedstocks, gas formation ceased during the temperature maintenance phase at 280°C. Thus, it can be assumed that the biomass conversion reactions reached their end point and that the maximum yields of solid, liquid, and gas products were achieved.

**FIGURE 4:**
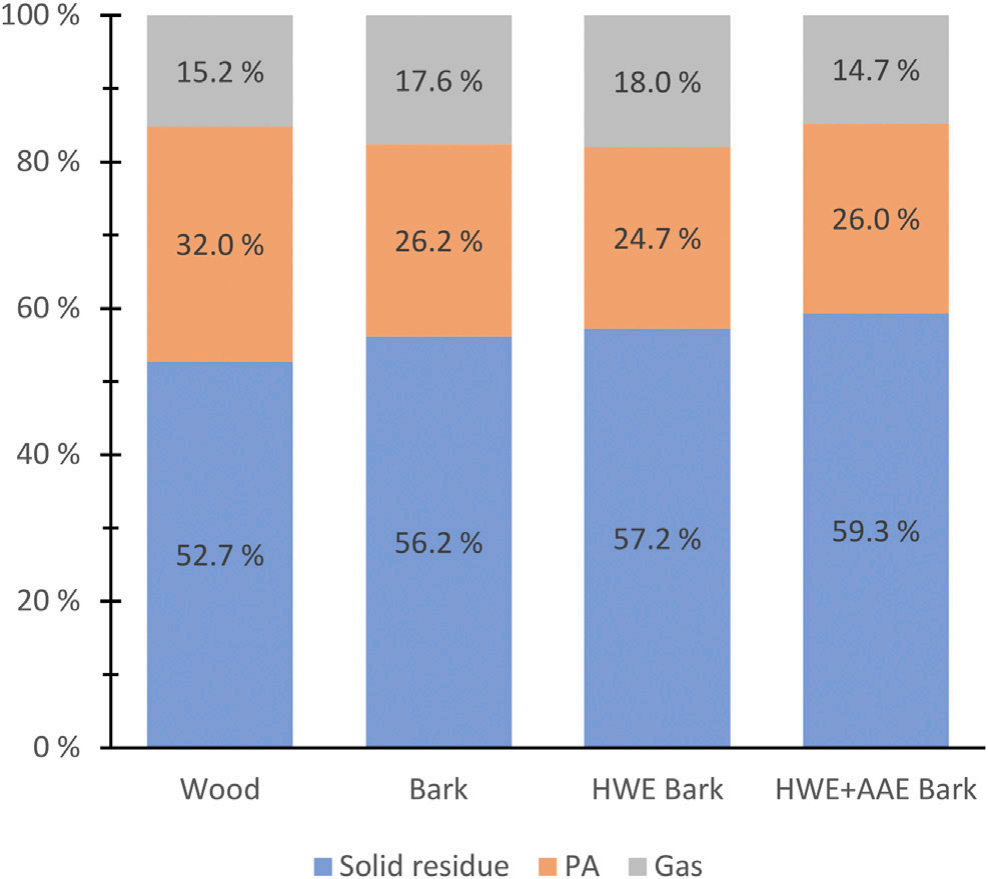
Solid pyroligneous acid (PA) and gaseous product yields (weight percent of feedstock dry weights) produced by torrefaction from hybrid aspen wood, bark, and pre-extracted bark masses.

Figure 4 shows the weight percent (wt.%) yields of the conversion products produced from wood, bark, and pre-extracted bark biomasses. The wood raw material was found to form the highest amount of PAs relative to the dry matter mass, although the total content of hemicelluloses was found to be lower than that in the bark. The higher wood PA yield can be expected as due to the higher xylan ratio of wood hemicelluloses (Prins et al., 2006). Pre-extractions of bark did not appear to have a notable effect on the total yields of PAs.

Wood biomass had the highest mass loss compared to bark and pre-treated bark biomasses during conversion (Figure 4). In bark torrefaction, HWE and HWE+AAE pretreatments appeared to decrease mass loss, although the differences were small (Figure 4). The effect of AAE treatment on bark mass loss was not expected, as various studies have found that alkali metals bound to organic sites in lignocellulosic biomass can significantly increase mass loss during torrefaction (Shoulaifar et al., 2016a; Shoulaifar et al., 2016b; Macedo et al., 2018; Zhang et al., 2018). The observed opposite result is interesting since bark AAE pretreatment is likely to cause Na residues in the biomass and would be expected to cause accelerated mass loss.

#### Pyroligneous Acid Composition

PAs contain precipitate (tar) and liquid phases, both of which were summed to give the total yield of PAs. Tar was separated from the solutions by centrifugation, and only the separated liquids were included in the subsequent herbicide and fungicide experiments to avoid the polyaromatic hydrocarbons (PAHs) in the tar fraction (Fagernäs et al., 2012). PAs obtained from HWE-treated bark contained the highest amount of tar (8.75 wt.%), followed by bark PA (4.09 wt.%), wood PA (1.85 wt.%), and HWE+AAE bark PA (1.29 wt.%).

**FIGURE 5:**
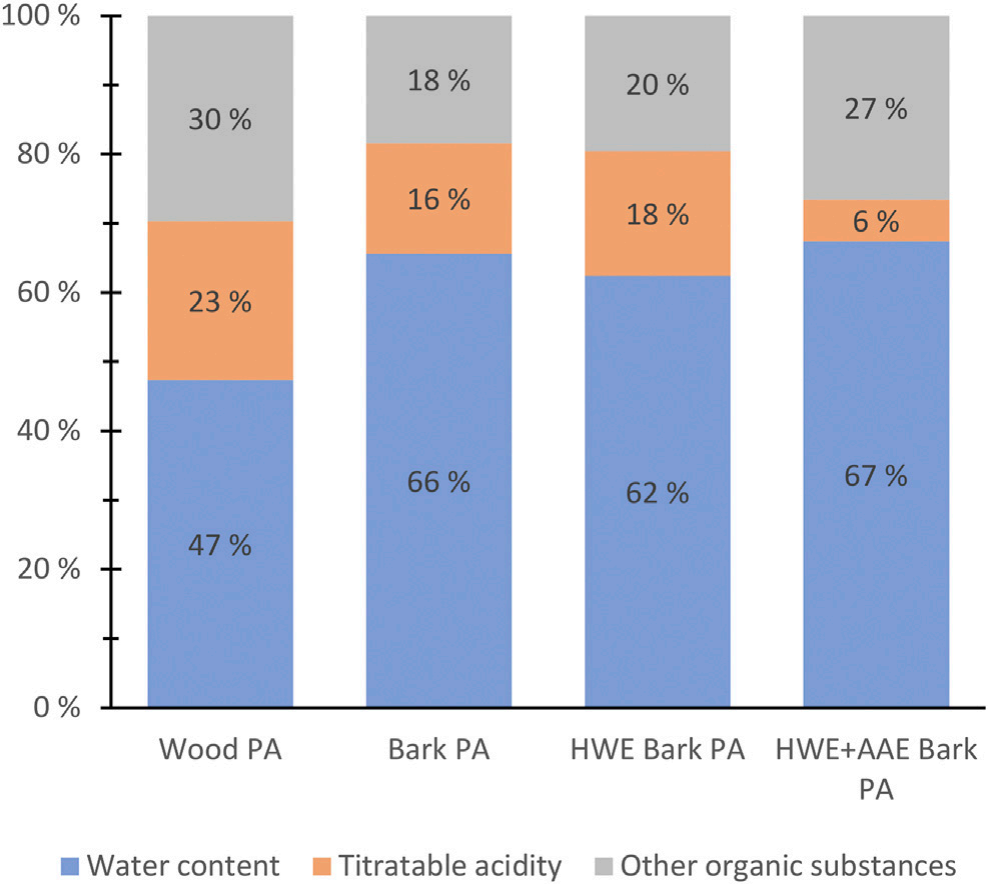
Basic chemical composition of pyroligneous acids (PAs) produced from wood, bark, hot water-extracted (HWE) bark or HWE+alkaline alcohol-extracted (AAE) bark of hybrid aspen.

Tar-free samples of bark PA were found to differ significantly from wood PA in their basic chemical composition (Figure 5). Bark pre-extractions also had a notable effect on the basic composition of PAs (bark PA *vs*. HWE bark PA *vs*. HWE+AAE bark PA) (Figure 5). The basic chemical composition of the PAs was determined by dividing the chemical fractions into groups of water content, acid content (CH_3_COOH *w*/*v* percent equivalent), and other contents of organic matter (Figure 5). Wood PA was found to contain a higher amount of acid, as well as organic matter, compared to the bark PA product. The higher acid content is explained by the higher xylan content of the wood (Prins et al., 2006). In terms of the bark feedstock pre-extraction processing, the most interesting finding emerged from the effect of HWE on the chemical composition of the produced PAs. The acid strength of PAs, as well as the proportion of organic matter, appeared to have increased because of HWE of the bark (bark PA *vs*. HWE bark PA) (Figure 5). However, following AAE, a significant negative effect on the acid content of PAs was shown, but the proportions of other soluble organic substances were clearly increased (HWE bark PA *vs*. HWE+AAE bark PA) (Figure 5). The decreased acid content of PAs can be explained by the alkaline extraction conditions that likely affected the hemicellulose xylan (Borrega et al., 2013).

**FIGURE 6:**
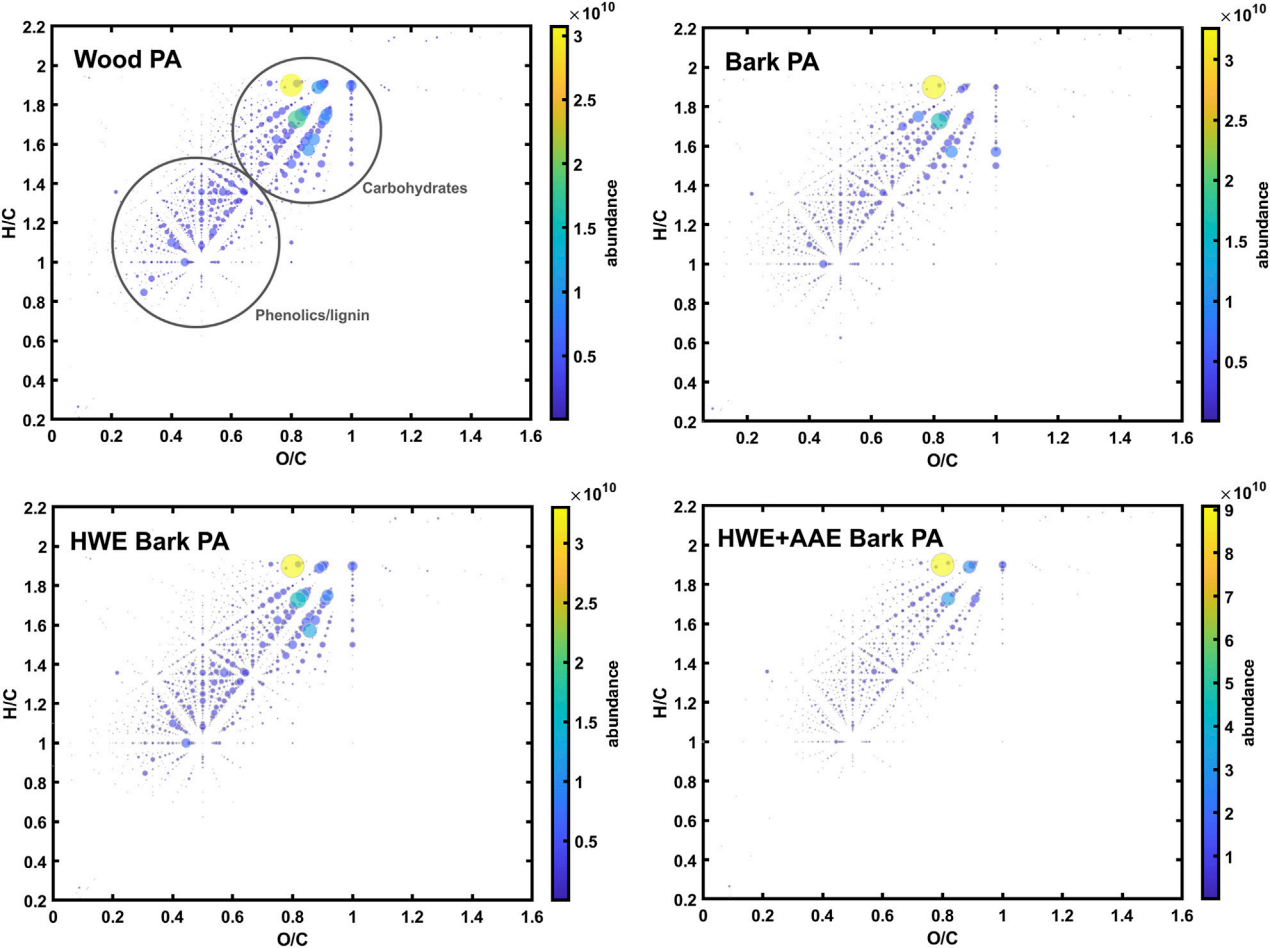
Van Krevelen diagrams for the compounds detected in different conversion distillates with negative-ion ion electrospray ionization Fourier transform ion cyclotron resonance (ESI-FT-ICR) mass spectrometry. The *dot size*/*color* represents the relative abundance of each detected compound.

The composition of the organic matter in the PA samples was further analyzed with negative-ion ESI-FT-ICR mass spectrometry, which specifically targets less volatile organic compounds present in thermochemical wood distillates (Zhao et al., 2020; Zhao et al., 2021), such as organic acids, phenolic compounds, and carbohydrates (sugars and anhydro sugars). Zhao et al. have previously shown that extractives, some phenolics, and hydrocarbons are enriched in the tar (i.e., water-insoluble) fractions, while the more polar oxygenates are enriched in the PA (i.e., water-soluble) fractions (Zhao et al., 2021). The van Krevelen diagrams for all the detected compounds in each sample are presented in Figure 6, which allow the overall chemical compositions to be compared. The compositions of the tar-free PA samples were similar, mainly comprising different phenolic compounds and carbohydrates (mainly hemicellulose-derived mono- and disaccharides) (Figure 6), consistent with the previous work of Zhao et al. (2021). However, the HWE+AAE bark PA had clearly lower amounts of phenolics and monosaccharides than the other samples. Due to the complex chemical nature of the samples, individual compounds were not further identified in this work. The smallest organic acids, alcohols, or furanic compounds were not efficiently ionized by ESI and needed to be analyzed by other means, e.g., GC-MS or photoionization combined with FT-ICR MS. These further analyses remain as a topic for future studies. The negative-ion ESI-FT-ICR mass spectra of the PAs are shown in the Supplementary Material (Supplementary Figure S1).

### Herbicidal Activity

**FIGURE 7:**
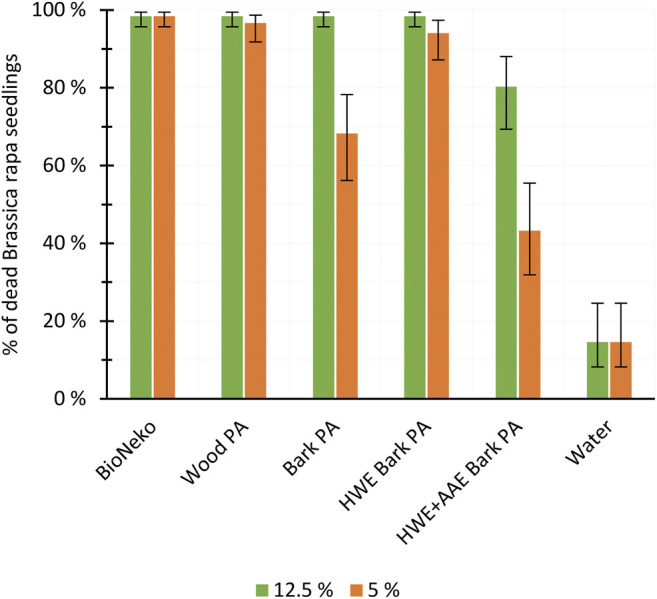
Herbicidal effectivity of pyroligneous acids (PAs) produced from wood and variously treated (*HWE*, hot water extracted; *AAE*, alkaline alcohol extraction) bark products (mean ± SD, *n* = 4) against *Brassica rapa* seedlings 7 days after the spraying compared to that of a commercial product (BioNeko) and water controls.

The herbicidal activity of the PAs produced from hybrid aspen fractions and pretreated bark masses was compared with that of the acetic acid-based commercial herbicide (BioNeko) and that of water. Seven days after the spraying, all PA solutions with 20% or higher concentrations eliminated 100% of the *B. rapa* seedlings, being as effective as the commercial herbicide (BioNeko), but each differing significantly from water (*p* < 0.05). At lower dilutions, differences between the PAs produced from various feedstocks were revealed (Figure 7): as 12.5% solutions, wood PA, bark PA, and HWE bark PA were as herbicidal active as BioNeko, but HWE+AAE bark PA was found to have a significantly reduced activity (HWE+AAE bark PA *vs*. all others, *p* < 0.05). At 5% dilutions (Figure 7), wood PA still retained its herbicidal activity, being as effective as the commercial herbicide (wood PA *vs*. BioNeko, *p* = 0.847). However, the herbicidal activity of bark PA was significantly lower than that of wood PA (5% bark PA *vs*. 5% wood PA, *p* < 0.001). HWE of the bark had a significant positive effect on the herbicidal activity of the PAs produced from the bark, as the performance of HWE bark PA was not statistically different from that of wood PA (HWE bark PA *vs*. wood PA, *p* = 0.919) or that of BioNeko (HWE bark PA *vs*. BioNeko, *p* = 0.274).

### Fungicidal Activity

**FIGURE 8:**
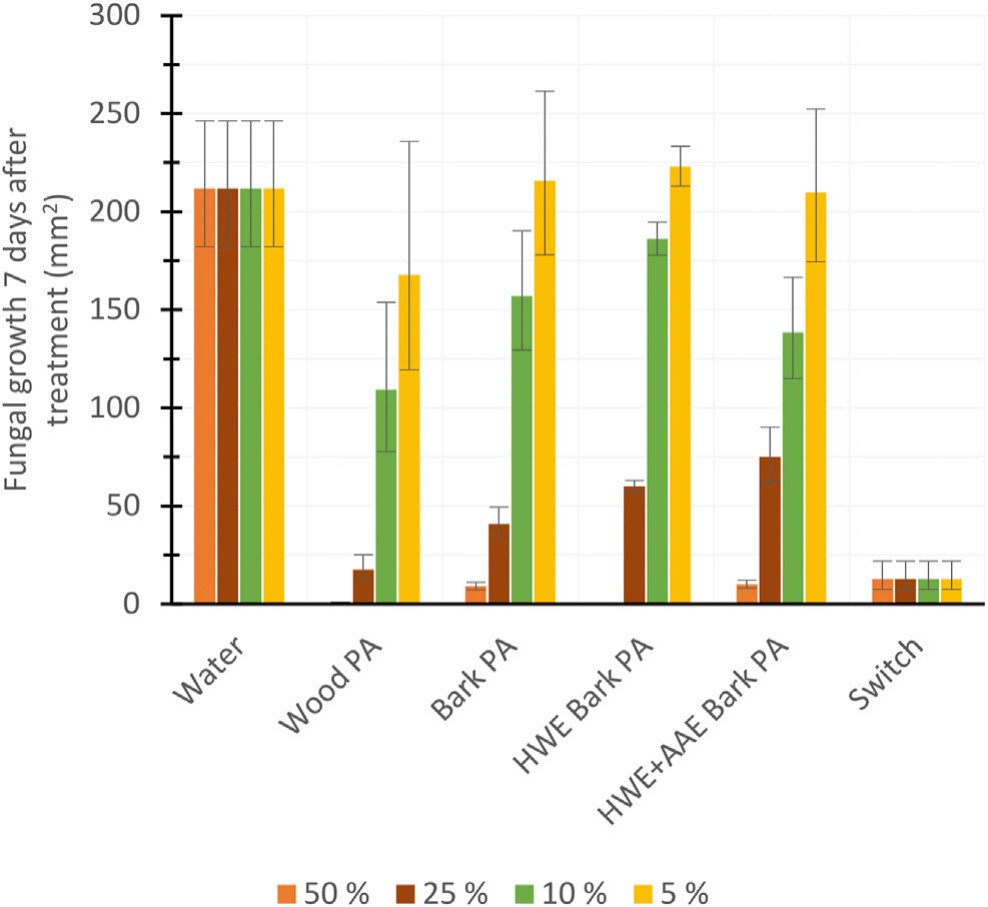
Fungicide potency of pyroligneous acids (PAs) produced from aspen tree wood, bark, or variously extracted bark (*HWE*, hot water extraction; *AAE*, alkaline alcohol extraction), commercial herbicide (Switch), or water measured *via* the inhibition of fungal (*Fusarium culmorum*) growth 7 days after the treatments (mean ± SD), *n* = 4.

**FIGURE 9:**
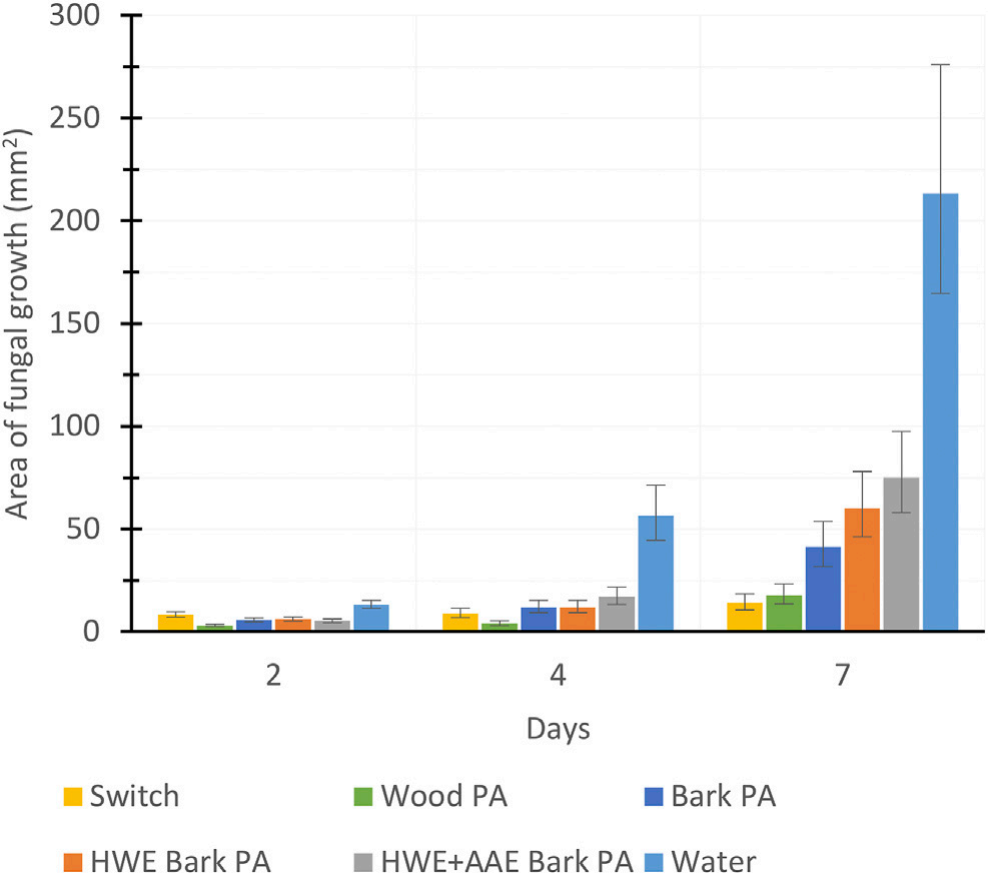
Fungal coverage area (in square millimeters) at 2, 4, and 7 days after treating the growth media with pyrolysis acids produced from wood, bark, hot water-extracted (HWE) bark, or HWE+alkaline alcohol-extracted (AAE) bark (mean ± SD, *n* = 4).

To investigate the fungicidal (*F. culmorum*) activity of PAs produced from hybrid aspen biomasses, we compared the antifungal efficiency of 50%, 25%, 10%, and 5% PA dilutions (Figure 8). In addition, the antifungal effect of 25% dilution of PAs produced from different biomasses was monitored for 7 days (Figure 9). Wood PA, bark PA, HWE bark PA, and HWE+AAE bark PA corresponded in 50% solutions to the commercial product Switch during the 7-day observation period, preventing *F. culmorum* from spreading almost completely (Figure 8). Dilutions of 25% of bark PA and pre-extracted bark PA showed a reduced antifungal effect, but wood PA still corresponded to Switch (wood PA *vs*. Switch, *p* = 0.866). It can be seen from the 25% dilutions in Figure 8 that the bark pre-extractions were found to have a negative effect on the fungicidal performance of the PAs produced. The growth area of *F. culmorum* was shown to be increased when comparing the growth inhibition performance of bark PA to that of HWE bark PA (*p* = 0.006). Comparison of the fungicidal performance between HWE bark PA *vs*. HWE+AAE bark PA showed that the effect appeared to have decreased further, although statistical difference was not found (*p* = 0.167). All PA solutions significantly lost their fungicidal activity against *F. culmorum* at 10% dilutions, and the activities no longer differed from water at 5% dilutions (all 5% PAs *vs*. water, *p* > 0.05). Figure 9 shows the *F. culmorum* growth inhibitory ability of 25% PAs over a 7-day follow-up period. After 4 days from treatments, differences in the fungicidal activity of PAs slightly appeared, but on day 7, only wood PA corresponded to the Switch product. Ascending growth trends suggest that the fungicidal performance of all tested treatment agents appeared to have decreased over the follow-up period, but this should be verified by extending the observation period.

### Current State of Pyroligneous Biopesticide Usage and Research

The use of PAs as herbicides, insecticides, and fungicides has a long tradition in many Asian countries (Yatagai et al., 2002; Tiilikkala et al., 2010; Hossain et al., 2015). PAs produced by slow pyrolysis have been proven to be effective against a wide number of pests (Yatagai et al., 2002; Lindqvist et al., 2010; Hossain et al., 2015), to have antifungal activity against pathogenic fungi and yeasts (Ibrahim et al., 2013; Oramahi and Yoshimura, 2013; Mashuni et al., 2020), and to induce systemic resistance to fungal diseases in plants (Kårlund et al., 2014). The results of our study agree with those of previous studies and extend the results to also concern PAs produced by torrefaction. However, we are not aware of any studies concerning the utilization of biomasses according to the cascade principle, which allows the separation of specific soluble and insoluble compounds before pyrolysis and biopesticide production. As HWE pretreatment even improved the herbicidal activity of bark PA, the cascading use, i.e., the separation and utilization of hot water-extractable chemicals before the production of PAs for herbicidal purposes, is possible. However, the pretreatments used decreased the fungicidal properties of the PAs. Consequently, it is the target organism that determines the usability of various pretreatments to produce biopesticides from wood biomass by torrefaction.

## Conclusion

The results of the study showed that both wood and bark biomasses of hybrid aspen (*Populus tremula* L. × *P. tremuloides* Michx.) are promising raw materials for the production of pesticide-active PAs on weeds and fungal diseases. PAs converted from wood, bark, HWE bark, and HWE+AAE bark were equally herbicidal active solutions at >20% and fungicidal active solutions at >50% with commercial products. The results are novel, as we showed for the first time that, along with the production of biopesticides, several other valuable products can also be separated from the bark, if the solution strengths of the herbicides and fungicides used are at least as described above. We further observed that HWE pretreatment improved the herbicidal activity of bark PA, which also supports the cascade utilization potential of the bark. No similar benefit was observed with alkaline alcohol extraction, but despite pretreatment, the PA did not completely lose its biopesticide potency. The results also showed that the fungicidal activity of PAs produced from hybrid aspen biomasses was lower compared to its herbicidal activity. This is evident in the differences of the activity of the PA dilution ratios tested. It can be summarized that utilization according to the cascade principle is possible by separating the tree biomass fractions into their own raw material sources and by using different separation extractions to form value side streams. Still, further research is needed to determine the market value and market entry potential of the products. Also, more detailed characterization of the chemical products produced from hybrid aspen biomasses awaits further research.

## Data Availability

The raw data supporting the conclusions of this article will be made available by the authors, without undue reservation.
