# Supplementary material for: Pyroligneous Acids of Differently Pretreated Hybrid Aspen Biomass: Herbicide and Fungicide Performance
Source: Front Chem. 2022 Feb 8;9:821806. doi: 10.3389/fchem.2021.821806 (PMC8861299; doi:10.3389/fchem.2021.821806)
Supplement: Supplementary file 1 [file DataSheet1.docx]

Supplementary Material

| 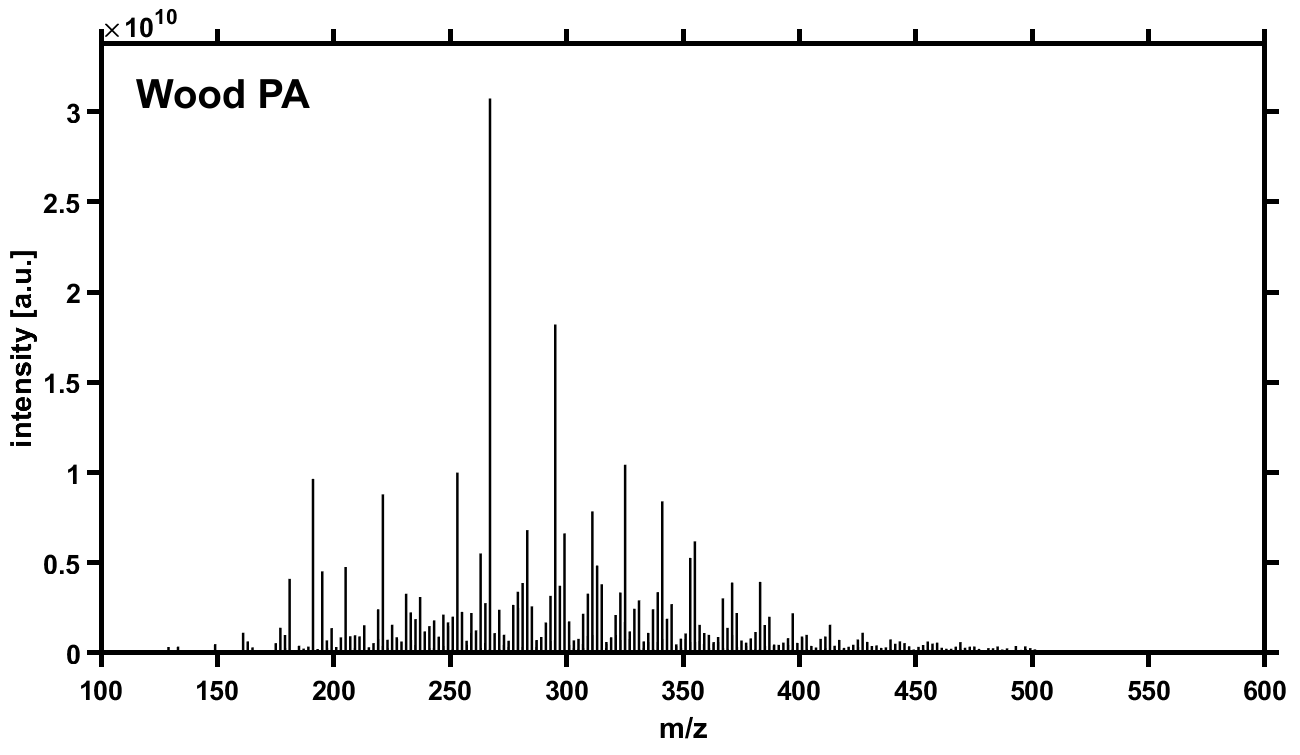 | 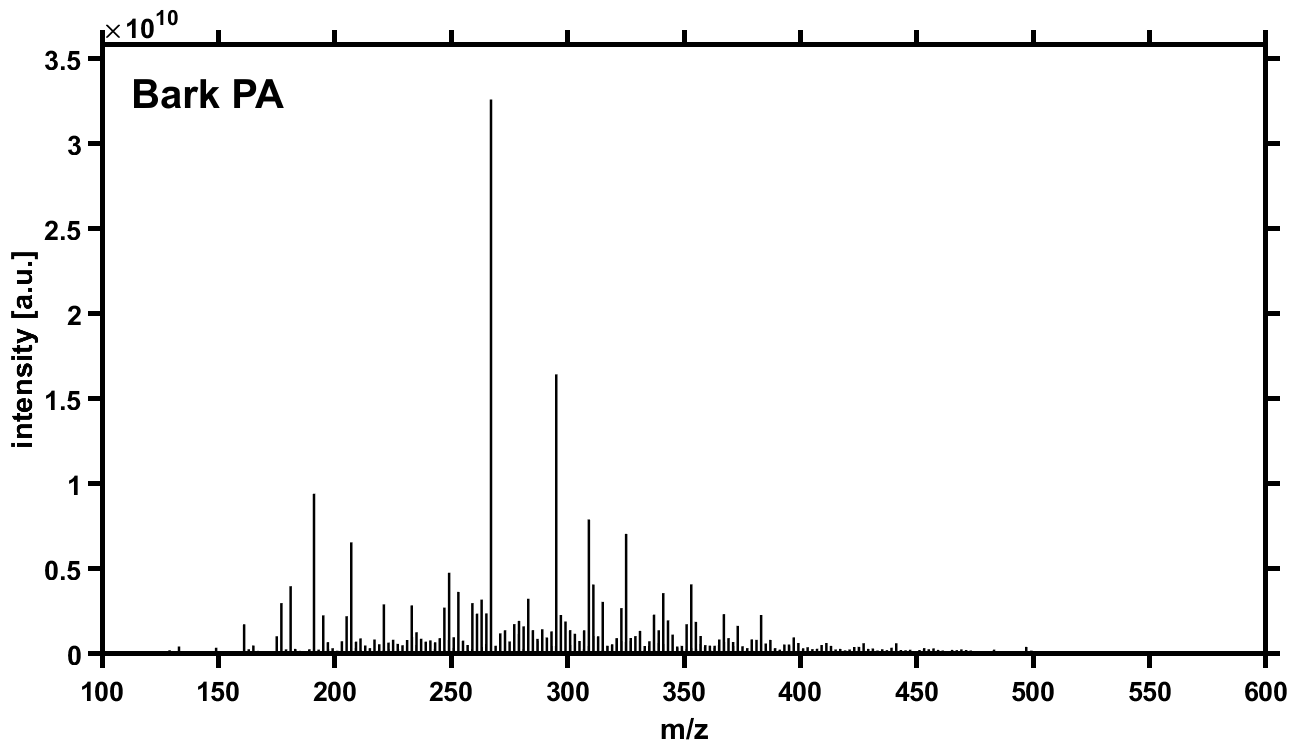 |
| --- | --- |
| 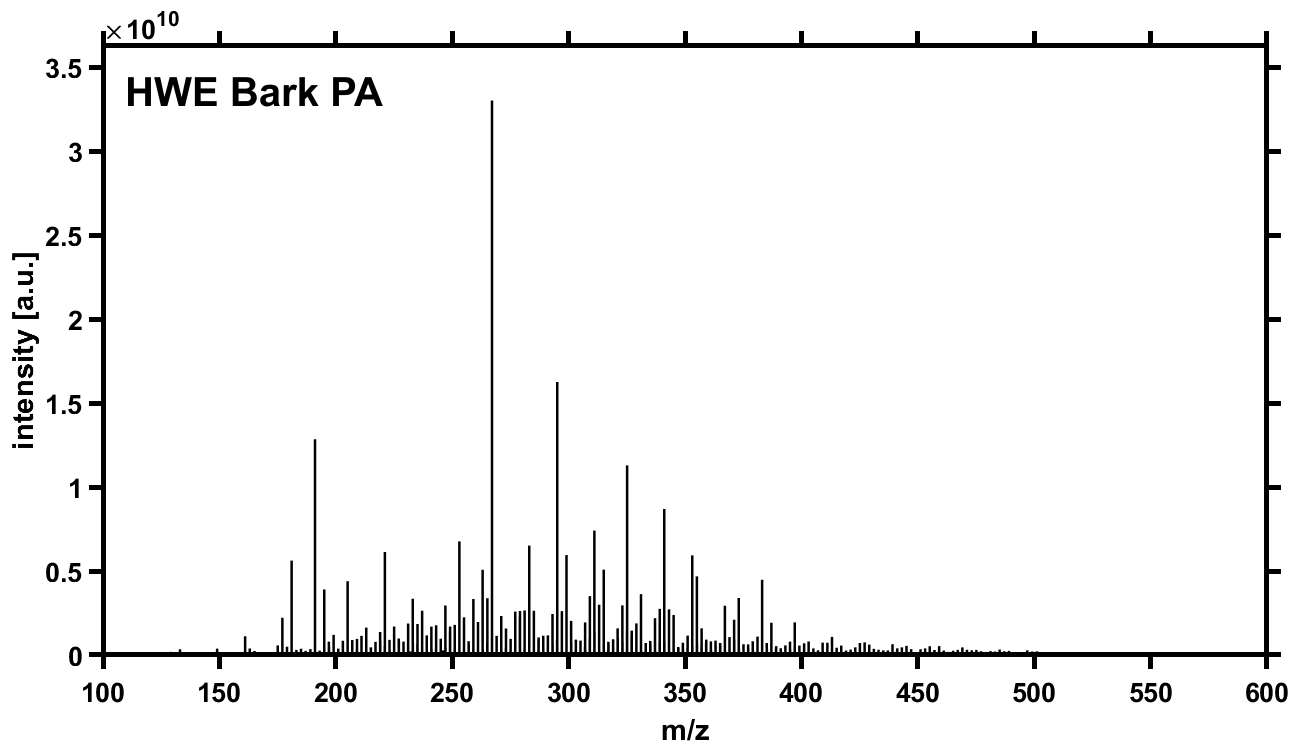 | 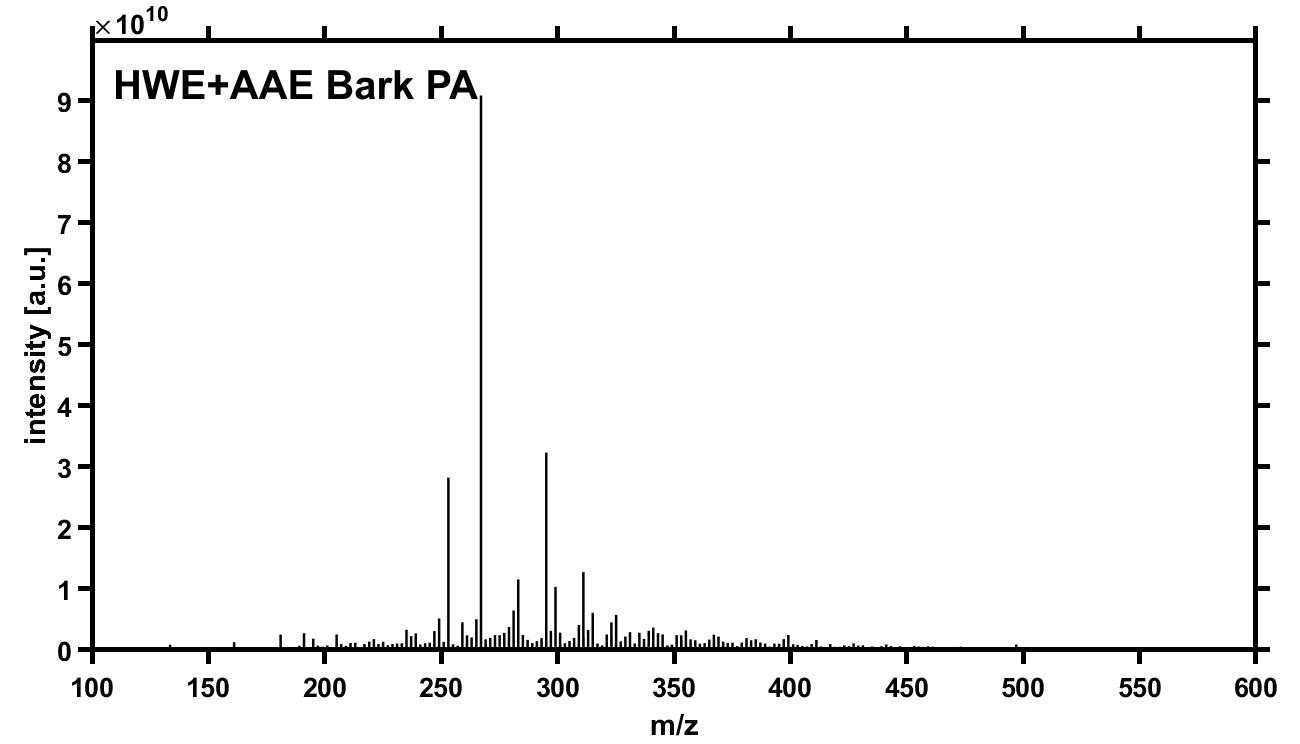 |

**Supplementary Figure 1.** Negative-ion ESI FT-ICR mass spectra of the wood, bark, hot water-extracted bark (HWE Bark), and hot water + alkaline alcohol-extracted bark (HWE+AAE Bark) pyroligneous acids (PA).
